# Framing and understanding the whole aspect of oral sex from social and health perspective: a narrative review

**DOI:** 10.12688/f1000research.108675.2

**Published:** 2022-03-09

**Authors:** Cennikon Pakpahan, Darmadi Darmadi, Agustinus Agustinus, Andri Rezano

**Affiliations:** 1 Andrology Study Program, Department of Biomedical Sciences, Faculty of Medicine, Universitas Airlangga, Surabaya, East Java, Indonesia; 2 Department of Internal Medicine, Faculty of Medicine, Universitas Sumatera Utara, Medan, North Sumatera, Indonesia; 3 Department of Biomedical Sciences, Faculty of Medicine, Universitas Padjadjaran, Sumedang, West Java, Indonesia

**Keywords:** oral sex, sexual behaviour, psychological well-being, pop culture, sexually transmitted disease, oral cancer, preeclampsia

## Abstract

Since thousands of years ago, oral sex has become part of sexual behavior among humans. Oral sex is considered taboo. Its taboo does not lie in the behavior, but its expression is deemed inappropriate. As technology becomes more sophisticated, human rights also stand out, leading to the disclosure of the practice in the 21 ^st^ century. The oral sex that is discussed on a large scale in media encourages people to express it as feedback whether within right or not. It all depends on the value of each people. We found that this sexual behavior is found everywhere regardless of religion, culture, and race.

Pop culture influences this behavior so much, it can be seen from music, movies, and television programs that provoke oral sex. Many motivations underlying this behavior include getting sexual pleasure for the sake of psychological well-being. But it is undeniable that this behavior is still controversial. It could be at risk of causing disease and, on the other hand, is reported to provide many benefits.

According to our theory, oral sex is not a new behavior crossing boundaries. It is just an old behavior that surfaces because of the factors that support it. This behavior, which is still considered taboo, has its disadvantages such as sexually transmitted disease and oral cancer but has also benefits such as preventing preeclampsia.

## Introduction

Oral sex is sexual activity with genital stimulation using the mouth, tongue, teeth, or throat. Currently, oral sex is frequent in both heterosexual and homosexual couples. Oral sex involves oral-vaginal contact (cunnilingus), oral-penile contact (fellatio), oral-anal contact (anilingus). ^1^ Besides that, Keating also stated that breast biting or licking was included in oral sex behavior. Oral sex has become a part of sexual behavior in society regardless of age. ^2^

Two types of oral sex widely accepted in society are “going down” activities, cunnilingus, and fellatio. The aim of the cunnilingus is stimulation of the clitoris. The cunnilingus can start with a kiss to the lower part of the stomach’s partner and then the thigh's side to the clitoris. ^3^ Meanwhile, fellatio or sucking (blow job) is an activity the penis will be stimulated by licking by his partner. His partner will lick the shaft of the penis, even the testicles. In fellatio, men can ejaculate, ejaculation can be done outside, or sometimes the partner will swallow ejaculate. This action sometimes causes discomfort and not necessarily for everyone but tasting this ejaculate becomes advanced satisfaction for the “sucker” or “kisser”. Fellatio and cunnilingus can be done simultaneously, known as the “69” position. ^3^ ^,^ ^4^ Moreover, it must be understood that ejaculate fluid still risks transmitting diseases. This should be considered and communicated to the partner when deciding this.

Oral sex can be considered an ancient activity from past manuscripts. As can be seen from the Hebrew, the Greeks to the Kamasutra manuals and the reliefs of several historical buildings depict this activity. Several moral, religious, medical, legal, and cultural factors influence the practice. Several cultures and religions give perceptive that oral sex is weird and unusual sexual behavior. ^4^ But currently, the trend of oral sex has increased prominently. The development of technology and media also supports this.

In general, people are less likely to consider oral sex has associated with negative sexual health outcomes and as “having sex”. The discussion of oral sex is important to discuss their associated risks but also the benefits. Recently, the prevalence of men and women who engage in oral sex has increased. The purpose of the present study was to determine role of pop culture towards oral sex, Did this increase due to freedom of expression? what were the motives behind oral sex activity and their associated benefit and risks including moral code and religion?.

## Methods

This study was to obtain a complete review and answer questions related to oral sex. We searched for articles and news related to oral sex in Pubmed, Google Scholar, and Emerald databases. For oral sex and pop culture, we use the keywords “oral sex” and “pop culture” or “hooking-up culture” or “film” or “music” or “literature”. To find out the number of cases of oral sex in our population, we used the keywords “prevalence” and “oral sex”. For information related to perspective and attitude related to oral sex, we searched “perspective” or “motives” or “attitude” and “oral sex”, and to review the risks and benefits of oral sex, we searched“benefits” or “risk” and “oral sex”. We reviewed relevant journals based on that keywords. We included original articles or opinions about oral sex that could answer our aims and wrote for each topic. As our attempt to review globally, there were no location/country restrictions included in this review.

## Results and discussion

### Oral sex and pop culture

Oral sex in this era is introduced in films and music videos, song lyrics, and literature. Several movies and music videos are not reluctantly labeled as hard-core and X-rated. Some scenes in the movie shown in public cinema presented men and women actively and even unstimulated in sexual positions, including oral sex. ^5^ The Brown Bunny’s Vincent Gallo, 9 Songs’s Michael Winterboom, Nymphomaniac vol 1& 2's Lars von Trier, Love's Gaspar Noe and many more movies are movies that promote explicitly oral sex in a graphic scene in the history of cinema. ^6^ Other than that in music, “Summer of 69” of Bryan Adams to the most recent Ariana Grande's song “34 + 35” are song which describes how oral sex activities are carried out by both men and women simultaneously with position 69. ^7^ ^,^ ^8^

These television programs and films also play a role in encouraging premarital sex behavior and uncommitted. ^9^ This trend is known as “hooking up”, this term refers to sex in an uncommitted and casual context. Oral sex is part of this “hooking up” trend. ^10^ ^,^ ^11^ Hooking up is depicted through films such as “No strings attached”, “Friends with benefits”, etc., or cable television program such as “MTV's Jersey Shore”. ^12^

Despite sexual expression between partners, oral sex can also be defined as a form of sexual harassment and violence. ^2^ Oral sex as a form of sexual violence can occur in heterosexuals and homosexuals. The rate of oral sex as an act of harassment varies widely. This suggests that investigations for sexual harassment do not have to focus on vaginal or anal genitals. However, tracing the oral organs is also necessary to confirm allegations of sexual misconduct. Evidence, for example, can be done by detecting sperm or seminal plasma in the victim's mouth in cases of fellatio or by performing penis swabs in cases of cunnilingus. ^13^

### The prevalence of oral sex of some countries around the world

Wylie conducted an extensive survey on 26,032 participants aged > 16 years in 26 countries from the Americas, Europe, Asia, and Australia. As many as 38% of the participants engaged in oral sex as a sexual activity. Oral sex has a very high prevalence among all sex orientation groups. ^14^ Meanwhile, data by Richters *et al.* reported 19,307 Australians aged 16 to 59 found 32% of the respondents had oral sex from their last sexual history. ^15^ According to D'souza *et al.,* there are differences in oral sex behavior compared to gender, age, and race. Oral sex behavior exists in married couples or adults and adolescents, and young adults. ^16^ In a report from the Child Trends Data Bank in 2015, adolescents aged 15-19 years, both boys and girls, reported having oral sex as much as 39% and 38%. Women said giving more oral sex than men. ^17^

In developing countries in Africa, such as Nigeria, it is reported that the influence of the media and the Internet affects a person's sexual attitudes and behavior. Out of 400 respondents, 352 people had oral sex. Some of them learned this behavior from the internet. ^18^ In Addis Ababa, Ethiopia, a study reported oral sex among high school adolescents in 3840. Adolescents who had practiced oral sex is 5.4% (190) person. Oral sex is closely related to the concept of best friends (AOR = 5.7; 95% CI 3.6-11.2) and having illiterate mothers (AOR = 11.5; 95% CI 6.4-18.5). ^19^ Besides that, oral sex behavior is one of the activities carried out and offered by sex workers. In some countries in Asia, the percentage of women who engage in oral sex varies 5% in Indonesia, ^20^ 16% in Thailand, ^21^ and 18% in India. ^22^ These numbers show that oral sex is not a new behavior in any world. Many of these behaviors are carried out even though it is a developing country and a sophisticated country; the difference is that technological developments accompanied by freedom of expression make this behavior a lot of popping up in the population.

### Perspectives and motives in oral sex

In adolescents and young adults, NBC (2005) reports that 40-47% of young people in USA who do oral sex do not need to worry about pregnancy, meet the right person, and feel the sense for the first time. Other reasons, many couples in serious relationships want to experience sexual pleasure but are not pregnant, ^23^ experience sex without using a condom, ^23^ ^–^ ^26^ maintain virginity, ^24^ ^,^ ^26^ answer curiosity about sexual activities, ^24^ then believed that oral sex has a low risk of STD transmission. ^23^ ^–^ ^26^

In teenagers, having oral sex is related to increased popularity, ^27^ improvement of relationships between teenagers, ^24^ ^,^ ^25^ engaging in sexual activity without commitment, ^23^ assumption that oral sex is not sex, ^23^ and also sex with someone is a generous behavior. ^27^ The same perspectives and motivations were also found in married couples. Holway and Tillman reported on the timing of oral sex in marriage among young adult couples in USA. Their report stated that women who were late or never performed oral sex reported being more satisfied with their relationships with their partners. The fundamental reason is they do not feel forced when having sexual intercourse and do not need to worry about the transmission of disease due to sexual relations. ^28^

Meanwhile, oral sex is behavior that risks transmitting various diseases. Teens and young adults even claim oral sex is less risky and more acceptable than vaginal sex. Sometimes they perform oral sex because they do not want contraception and protection while having sex. ^29^ This fact shows the need for health workers and counsellors who provide the correct perspective in education or sex counselling. ^28^ ^,^ ^29^

Penhollow *et al.* also conducted a study on 408 students in USA. They asked students' involvement in religious activities, frequency of worship attendance, their feelings about religion, their perception of God in sex and then looked at their participation in several sexual activities such as oral sex. ^30^ Results indicated that religiosity variables, mainly frequency of religious attendance and religious feelings, were significant predictors of sexual behavior such as oral sex. ^18^ The religious factor was reported as one of the reasons a person does not engage in oral. ^16^ Apart from that, another influencing factor in higher education. ^28^

### Attitudes in oral sex

Attitudes towards oral sex tend to vary in population. The study surveyed 8600 people aged 16-64 years in Australia regarding their sexual activity. The analysis results stated that oral sex is one of the mandatory activities in sex activities besides kissing, cuddling, and vaginal intercourse. About 74% of their respondents reported that women want stimulation over their genitals orally, and 70% stimulate their partners' genitals orally. ^31^ This proves that genital mouth stimulation is widespread in married couples. ^32^

Men at risk for adultery are more likely to engage in oral sex. ^33^ Oral sex can be used to detect infidelity, material retention behavior, and orgasm with sperm retention. Oral sex, which is used as s detector for adultery, is indicated, for example, in cunnilingus activity; one study states that men are still likely to feel and smell their rival's semen around the vagina related to their previous sexual activity. ^34^

Many studies have reported that couples during oral sex mostly do not use condoms. However, Auslender *et al.* from USA reported different results. The results were all in young adults when oral sex took protective measures to reduce the process of transmitting diseases such as *Herpes simplex* such as microbicide surrogate products. ^35^

In 2015, a study in Northeastern University, USA enrolled 346 men and women regarding their expressions and attitudes towards oral sex, either receiving or doing for the first time. They address various expressions: happy, fearful, indifferent, strange, disgusted, surprised, relieved, proud, and sad. The most common expression for oral sex recipients was feeling happy (38.7%). Interestingly, 10.8% thought this behavior was disgusting, and 8.2% thought this was strange. In another case who did oral sex, dominant among them felt fearful as much as 17.1%, 15.8% felt indifferent, 2.8% felt disgusted, and only 12% felt happy doing it. ^36^ Others reported on a focus group discussion study involving women with experiences of fellatio and cunnilingus. They stated a feeling of good emotional vulnerability when engaging in oral sex on their partners. ^37^

During oral sex, the aim is to orgasm for the partner. Richerts *et al*. (2006) conducted a study on a large population in Australia, 50% of women experience orgasm during vaginal intercourse, but when intercourse is added with cunnilingus activity, 73% will experience orgasm. Oral sex strengthens the increased incidence of orgasm in partners, especially in women. ^15^

### Oral sex risk

Many reports have stated that oral sex can be local infections, rather a cunnilingus, fellatio, or anilingus. Pathogens that are often transmitted through oral sex are viruses ( *Herpes simplex viruses*, *Hepatitis virus*, *Human papillomaviruses*, and less frequently HIV) and bacteria (mainly *syphilis, gonorrhea*, group B *Streptococcus*). ^38^ ^,^ ^39^ In addition, infections such as *Molluscum contagiousum*, *Candidiasis, Epstein Barr viru*s, and *Aspergillosis* can also be spread through the process of oral sex. ^40^

Gonorrhea infection is possible in oral sex. Sex workers with inconsistent condom use for oral sex were reported 17.1 times more likely infected (95% CI: 8.0 ± 36.5) than consistent condom users to develop pharyngeal gonorrhea. ^41^ HSV-1 is transmitted through genital and oral contact. This lesion is one of the most prevalent lesions among women, even young. ^42^ ^,^ ^43^ HSV-1 is more likely to spread to the female genital organs than male genital organs. ^44^ For young people, acquiring a lifelong recurrent infection such as genital herpes is not only an unfortunate surprise diagnosis, it can also invoke anxiety, guilt, and social-sexual isolation. ^45^ ^,^ ^46^

Unusual infections have also been reported after oral sex. The incidence of pharyngitis with Trichomoniasis vaginalis was reported in men who routinely had oral sex with their partner who had vaginal Trichomoniasis. ^47^ So far, it is infrequent for Trichomoniasis to spread by mouth and cause a local infection. In addition, parasites such as Toxoplasma gondii is found in ejaculate fluid, possibly spread through oral sex. ^48^ ^,^ ^49^ There may be a positive association or association between Toxoplasma transmission and oral sex behavior, especially fellatio. However, this condition depends on the activity of fellatio, whether it will be sperm swallowed or not.

A severe infection has been reported by Froissart *et al*. They reported two cases of severe infection that occurred through oral sex activity. The first case complained of fever, pubic erythema, penile edema, and cellulitis and the second case complained of genital bubo, foreskin edema, fever, glans erosion, and cellulitis. Both of these cases occurred after performing fellatio activities with their sex partners. In this case, the suspicion of the transmission process was an abrasion of the penis during traumatic oral sex. Cellulitis of the pubis is an infrequent case. The trauma of the genital organs due to the bite of the partner is presumed as a port de entry. ^38^

Severe soft tissue infections such as Fournier's gangrene were also reported by Lutz and Gerber (2020) through oral sex with commercial sex workers. This male patient experienced swelling and sloughing of the skin of his penis after reportedly engaging in oral sex. During intercourse, his partner nicked the shaft of the penis using her teeth. ^50^ Takenouchi *et al.* also reported a rare infection, Lemierre syndrome, in a 58-year-old man after oral sex with a sexual partner two days previously. ^51^ Lemierre syndrome is characterized by thrombophlebitis of the internal jugular veins and bacteremia caused by anaerobic organisms following a recent oropharyngeal infection. ^52^

Cunnilingus should be avoided in pregnant women. Hosseini and Hunt reported a case of *Streptococcus mitis* Chorioamnionitis in a 43-year-old pregnant woman who had oral sex with his partner. This infection occurred ten days before contractions after dental scaling and oral sex. ^53^ The same case was also reported by Gherman *et al.* in Australia that infected by *Streptococcus viridans* after cunnilingus. ^54^ Both *Streptococcus mitis* and *Streptococcus viridans* are normal flora in the oral cavity.

WHO's guidelines for adolescent sexual and reproductive health and rights in 2018 recommends giving antibiotic prophylaxis to children who experience sexual abuse with the suspicion of one of which is oral sex, WHO considers that oral sex by unknown offender has a significant risk of sexual transmitted disease. ^55^ According to Barbara *et al*., oral sex was the riskiest behavior than vaginal and anal sex. Cunnilingus has an OR of 2.199 and fellatio of an OR of 2.756 to have infected. This study reported that people who performed oral activities have higher risks associated with STD/HIV transmission than those not. ^56^

Other serious risks that arise due to oral sex behavior include human papillomavirus infection, which causes oral squamous papilloma, oral verruca vulgaris, condyloma acuminata, focal epithelial hyperplasia, epidermoid carcinoma, and oropharyngeal squamous cell carcinoma. ^57^ ^–^ ^59^ One study from Indonesia proves a strong correlation between the activity of oral and anal among homosexuals and HPV infection (OR 6.854). ^60^ According to Brown *et al*., women who had more than three times oral sex with their partners in the previous month had a higher risk of developing oral HPV than those who had less. ^61^

### Oral sex benefits

Meuleman *et al.* reported in Netherlands that oral sex might be a protective factor in recurrent miscarriage cases. This study was conducted on 97 women who experienced unexplained consecutive miscarriages. Recurrent miscarriage cases occur due to an imbalance of immunity in the embryo's implantation into the endometrium. ^62^ Koelman *et al.* from The Netherlands also stated that exposure to oral sex could reduce the incidence of preeclampsia. ^63^

Pittrof *et al.* performed a study on 619 women engaged in oral sex in USA. They have a lower risk of developing endometriosis and pelvic inflammatory disease than their counterparts. The assumption that oral sex can be a protective factor is that endometriosis and PID require adaptive immunity in the lymphoid system. ^64^ Meanwhile, the oropharynx is a channel that is rich in lymph channels, thereby facilitating the stimulation of adaptive immunity. Primed lymphocytes in the nasopharyngeal tract will be found later in the endocervix. ^65^ In addition, it is possible to induce genital T-cells and B-cells through sub-lingual immunization. ^66^

Testosterone also affects the libido of women. Testosterone in semen can logically affect the libido of the sexual partner. Women who did not use condoms at the time of intercourse had more sexual intercourse than women who did. ^67^ But there are no specific reports in the case of women who swallowed semen. Semen also had an antidepressant effect, such as serotonin. It has been reported that women whose partners used a condom during intercourse were more likely to be depressed than those who did not use it. ^67^

Some interesting substances are opioids such as endorphin, enkephalin, and other cytokines also present in semen. These substances affect sperm motility, such as endorphins and calcitonin. ^68^ Endorphin and enkhapalines function to reduce anxiety and induce analgesia and drowsiness. ^69^ Likewise, with oxytocin, oxytocin is a hormone that plays a role in increasing bonding and intimacy in partners. This hormone impacts penile erection and female orgasm. ^70^ However, in the case of semen ingestion, the activity levels of these substances will be significantly reduced in the blood due to the absorption, distribution, and metabolism processes.

Besides that, several benefits are felt during oral sex, such as satisfaction in intercourse and emotional or pleasure in intercourse. A study on 410 women who had oral sex in USA and Germany showed that their male partners delay orgasm so that the intercourse time is longer. ^71^ Oral sex is a positive activity that provides sexual satisfaction and relationships. ^72^ ^,^ ^73^

Another benefit of oral sex is sperm retention by male partners. ^74^ Cunnilingus is considered one of the activities that help women experience orgasm. ^15^ Reports show that oral sex helps sexual activity last longer than usual. ^75^ Another study from USA reported that 233 men who had oral sex with their partners would spend more time having sex with them, doing more copulatory behavior that replaced semen, and reported greater sexual arousal. ^33^ Men engaging in oral sex with their partners as part of strategy provide a more comprehensive range of benefits. Men who are higher in consideration are more likely to benefit their partner. ^75^

## Future directions and conclusion

All agree that “bedroom activities” are private and personal matters. We must understand that one's sexual fantasies and bed activities are a social and moral responsibility. Sometimes it's hard to judge that a “certain bed activity” is right and wrong. If it is closed among couples and both enjoy and accept each other, it's fine to choose what kind of sexual preference. All need to be returned to the individual reason they chose and did that sex activity.

The research we reviewed reported a lot of oral sex activity in the USA and Australia but very few in the Middle East and Asia where western countries are known to be more liberal in sexual behavior. Although considering the perspectives, motivations, and attitudes behind oral sex, some of the reasons and arguments are worth understanding. Oral sex is used as a medium to enjoy each other and show affection for each other but love and sex are two different things. Expression of love does not always end with sex. In the triangular theory of love, sex is included in the passion which is only one part of the love component. ^76^ So the expression of love which is translated into sexual behavior is not always justified, especially when it gives rise to preferences such as paraphilias. ^77^ Things like this are often found in married couples. However, not all couples accept all these behaviors, therefore it is necessary to re-communicate what their respective sex preferences are like.

Oral sex has been known for a long-time clash with the moral code and religious values of each people. Although it is an ancient sex behavior, oral sex is not accepted by all circles. The religious factor is one of the factors that shapes people's cognition to consider whether it is right or not. ^18^ Quoting several religious views regarding oral sex, in Christianity, sex is something sacred and full of commitment. ^78^ Bed life in the Bible is described a lot in “the book of “Song of Songs”, this section shows that sex is something intimate and sacred between couples. ^79^ Ed. Wheat and Gaye Wheat in their book “Intended for Pleasure” state that oral sex is not explicitly found in the Bible, but Wheat argues that back to the basic, understanding that everything that is not good and inappropriate for the body is not worth doing. Every human being is responsible for their own body to glorify God as written in 1 Corinthians 7:3-5 and 1 Corinthians 3:6. ^80^

In Islamic religion, Chawki stated in his article, that oral sex is a disgusting activity and should not be done, but some views state that this is not explicitly stated. ^81^ Another opinion said that oral sex is something that can be done as long as it is done in the corridor of husband and wife as written in Q.S. Al-Baqarah: 223. ^82^ Some views also state that except for those explicitly disallowed (such as anal intercourse, oral sex, and sadomasochism), the married couple may pick any practical and mutually agreeable type of intercourse or other sexual activity. ^83^ In Buddhism, sexual misconduct is exemplified in the *Sutra of the Upsaka Precepts*, such as sex at the wrong time, wrong place, incorrect partner, virgin or another man's wife; or if he engages in sexual self-gratification. ^84^ Values like this will affect cognition and are returned to each individual. We agree values like these are important in shaping their perception of (oral) sex. However, a strict understanding of religion also affects a person's rigidity in understanding sex, so sometimes many people fall into disorders such as sexual aversion or sexual disgusting because they view sex as a sin. ^85^

Comprehending that oral sex is not always safe, everyone should have right “sex education” to be more careful about having oral sex. Especially teenagers and young adults who are not married yet, oral sex that is done just for the sake of popularity, satisfying curiosity is not justified because the risks outweigh the benefits. According to our analysis, it turns out that pop culture and technology are the factors that contribute more to oral sex behavior, although the results of many studies are obtained in countries such as the USA and Australia, we also find these factors in countries such as Nigeria and Ethiopia. ^18^ ^,^ ^19^ So, parents are needed to direct and monitor teenage about sex. ^86^

For couples, sometimes pleasure considerations should be put aside rather than safety and health. If oral sex only aims to get pleasure, couples must understand that pleasure does not always have to be with sex, especially oral sex. Couples must understand that there is a “sensate focus”, an activity that can deliver pleasure without having sex. ^87^ But on the other hand, the health benefits obtained through oral sex such as immune modulation in preventing recurrent miscarriages and preeclampsia deserve to be considered and even investigated further. In the end, oral sex was returned to their respective views with care.

## Data availability

No data are associated with this article.
