# Framing and understanding the whole aspect of oral sex from social and health perspectives: a narrative review

**DOI:** 10.12688/f1000research.108675.3

**Published:** 2022-07-21

**Authors:** Cennikon Pakpahan, Darmadi Darmadi, Agustinus Agustinus, Andri Rezano

**Affiliations:** 1 Andrology Study Program, Department of Biomedical Sciences, Faculty of Medicine, Universitas Airlangga, Surabaya, East Java, Indonesia; 2 Department of Internal Medicine, Faculty of Medicine, Universitas Sumatera Utara, Medan, North Sumatera, Indonesia; 3 Department of Biomedical Sciences, Faculty of Medicine, Universitas Padjadjaran, Sumedang, West Java, Indonesia

**Keywords:** oral sex, sexual behaviour, psychological well-being, pop culture, sexually transmitted disease, oral cancer, preeclampsia

## Abstract

Historically, oral sex is part of human sexual behaviour even though some consider it taboo, and its expression is deemed inappropriate. Human rights also stand out as technology becomes more sophisticated, leading to its practice disclosure in the 21 ^st^ century. The large-scale oral sex discussion in media encourages people to express it, and there is feedback on whether it is right or not. It all depends on each person’s values. We found this sexual behaviour occurs everywhere regardless of religion, culture and race.

Pop culture influences this behaviour considerably. It can be seen in music, movies and television programmes that allude to oral sex. Numerous motivations underlying this behaviour include sexual pleasure for the sake of psychological well-being. It is undeniable that this behaviour is still controversial. There is a risk of it causing disease, but it reportedly provides many benefits.

Oral sex is not a new behaviour crossing boundaries, according to our theory. It is an old behaviour that has been highlighted because of the factors that support it. This behaviour, still considered taboo, has its disadvantages, such as sexually transmitted disease and oral cancer, but also has benefits, such as preventing preeclampsia.

## Introduction

Sexual activity with genital stimulation using the mouth, tongue, teeth or throat is called oral sex. Both heterosexual and homosexual couples engage in oral sex, including oral-vaginal contact (cunnilingus), oral-penile contact (fellatio), and oral-anal contact (anilingus). ^1^ Keating also stated that breast biting or licking was included in oral sexual behaviour. It is part of sexual behaviour in society regardless of age. ^2^

Cunnilingus and fellatio are the two types of “going down” activities in oral sex widely accepted in society. Clitoral stimulation is the aim of cunnilingus. It can start with a kiss on a partner’s lower part of the stomach, the thigh, and then to the clitoris. ^3^ Meanwhile, an activity where a partner stimulates the penis through licking or sucking (blow job) is called fellatio. The partner will lick the shaft of the penis, even the testicles. Men can ejaculate outside, or sometimes the partner will swallow the ejaculate in fellatio. This action sometimes causes discomfort and is not necessarily for everyone, but tasting ejaculate becomes advanced satisfaction for the “sucker” or “kisser”. Fellatio and cunnilingus can be simultaneous, known as the “69” position. ^3^ ^,^ ^4^ However, ejaculate fluid has the risk of transmitting diseases, which should be communicated to the partner before engaging in the behaviour.

Oral sex is considered an ancient activity based on past manuscripts from the Hebrews, the Greeks, the Kamasutra manuals, and the reliefs of several historical buildings depict this activity. The practice is influenced by several moral, religious, medical, legal and cultural factors. Several cultures and religions consider oral sex weird and unusual sexual behaviour. ^4^ The trend of oral sex has increased prominently. The development of technology and media also supports this. ^5^

Vyomakesisri et al. described a grouping of beliefs, concepts, attitudes, points of view, images, or whatever the majority of people like pop culture as a common denominator. ^6^ This term has been widely used on various platforms, including music, literature and film, since the 1980s. ^6^ Sex in pop culture has much to do with people, especially young people. The “hooking-up” culture is sexual behaviour that is part of pop culture, and this is widely exhibited through technological developments such as movies and variety shows. ^5^ Undeniably, “sex with strangers” and “one-night stands” encourage people to explore sexual activities that are not usually like oral sex; this has led to an increase in the incidence of oral sex from time to time. ^5^ Many people have raised concerns about increased oral sex, including health care professionals, researchers, school personnel and others, especially among adolescents, although there is little factual data. ^7^ Not much different in the elderly, oral sex may be more common among older adults more recently. ^8^

The reasons that increase adolescents have oral sex vary widely, from improved social standing to health reasons. It begins with improved self-esteem and argues that oral sex is not “sex”. On the other hand, this behaviour supports their social existence. ^9^ Another opinion says oral sex has a lower risk of STIs than vaginal sex. ^9^ Older couples argue that oral sex provides a variety of sexual activities in a relationship, which significantly benefits relationship quality. ^8^ However, oral sex behaviour increases adolescents’ incidence of sexually transmitted infections (STIs) when viewed from a health aspect. ^10^ ^,^ ^11^ In addition, moral and religious views on oral sex make people consider whether oral sex is appropriate or not. However, the positive impact on the recipients of oral sex reportedly confers immune benefits, such as preventing preeclampsia. ^12^

People are generally less likely to consider that oral sex is associated with negative sexual health outcomes and as “having sex”. This review focuses on trying to understand oral sex from various perspectives, both from social and health aspects as follows: (1) How is oral sex related to today’s pop culture; (2) The prevalence of oral sex based on existing research reports; (3) Perspectives and motivations underlying oral sex behaviour, and social and cultural influences; (4) Aspects of oral sex health, along with the benefits obtained; (5) Religious view and moral issue on oral sex.

We take a narrative review approach in writing this article. There is no particular protocol in narrative review writing referring to Ferrari, such as a systematic review. ^13^ Therefore, there are no specific restrictions on the recruitment of eligible articles to be included in the study of this article, including language, year and location. The article deserves to be used as a reference in this review if the article is written with scientific principles.

## Oral sex and pop culture

The phrase “pop culture” entered the public language in the 1980s. We would use the word “popular” to describe things like the finest music playlists, pop art or the best- or top-selling novels in the past. ^5^ Pop culture is engaging, fascinating and essential to our daily lives. It is present everywhere, including sports, celebrities, video games, music, television, apps, movies, concerts or stage plays. ^6^ Oral sex behaviour is also introduced through this platform explicitly from year-to-year. Speaking openly about oral sex has become mainstream in modern popular culture and has also woven itself into the fabric of social mores and customs. ^14^

Films, music videos, song lyrics and literature introduced oral sex in this era. Several movies and music videos are not reluctantly labelled as hard-core and X-rated. Some scenes in movies shown in public cinema presented men and women actively in sexual positions and even unsimulated sex, including oral sex. ^15^ The Brown Bunny, 9 Songs, Nymphomaniac Vol 1& 2, Love and many more are movies that promote explicit oral sex in cinematic graphic scenes. ^16^ In music, “Summer of 69” by Bryan Adams and most recently Ariana Grande’s “34 + 35” are songs that describe how oral sex activities are carried out by both men and women simultaneously with position 69. ^17^ ^,^ ^18^

Television programmes and films also play a role in encouraging premarital and uncommitted sexual behaviour. ^19^ This trend is known as “hooking up” and refers to sex in an uncommitted and casual context. Oral sex is part of the “hooking up” trend. ^5^ ^,^ ^20^ Hooking up is depicted in films such as “No strings attached”, “Friends with benefits”, etc., or cable television programmes such as “MTV’s Jersey Shore”. ^21^

Popular culture has evolved into a source of sex education but is rife with (inaccurate) depictions of sexuality. Numerous popular depictions imply that casual sex, sometimes known as “hooking up”, can be biophysically and emotionally pleasurable and take place without “strings.” ^5^ In a “hooking up” culture, a heterosexual or homosexual person agrees to have sexual activity (kissing, touching, oral sex, vaginal sex, or anal sex) without any romantic relationship and commitment. Both do it solely for self-gratification. ^5^ Reiber and Garcia reported that of those who were influenced by a “hooking up” culture, 36% had oral sex, and 35% received oral sex. ^5^ In addition, because they want to get the sensation of having sex without using protection, those affected by the “hooking up” culture reported 0% condom use. ^22^ Oral sex is increasingly being reported because it facilitates orgasm as their sexual experience progresses, especially in those who perform cunnilingus. ^23^ Pornography and unsimulated sexual scenes as part of popular culture support oral sex behaviour. Oral sex is a common sexual behaviour in the modern era. Men and women have reportedly performed oral sex at least once. ^24^

Oral sex can also be defined as a form of sexual harassment and violence despite it being a form of sexual expression between partners. ^2^ Oral sex as a form of sexual violence can occur in both heterosexuals and homosexuals. The rate of oral sex as an act of harassment varies widely. This suggests that investigations for sexual harassment do not have to focus on vaginal or anal genitalia. However, tracing oral organs is also necessary to confirm allegations of sexual misconduct. Evidence can be secured by detecting sperm or seminal plasma in the victim’s mouth in fellatio or by performing penis swabs in cases of cunnilingus. ^25^

## The prevalence of oral sex in some countries around the world

Wylie conducted an extensive survey on 26,032 participants aged > 16 years in 26 countries from the Americas, Europe, Asia and Australia. As many as 38% of the participants engaged in oral sex as a sexual activity. Oral sex has a very high prevalence among all sexual orientation groups. ^26^ Meanwhile, data by Richters et al. reported that among 19,307 Australians aged 16 to 59, 32% of the respondents had oral sex from their last sexual history. ^27^ There are differences in oral sex behaviour compared to gender, age and race, according to D’souza et al. Oral sex behaviour exists in married couples, adults, adolescents and young adults. ^28^ Adolescent boys and girls aged 15–19 years in a report from the Child Trends Data Bank in 2015 reported having oral sex as much as 39% and 38%. Women gave more oral sex than men. ^29^

It is reported that media influence and the Internet affect a person’s sexual attitudes and behaviour in developing African countries, such as Nigeria. Out of 400 respondents, 352 people had oral sex. Some of them learned this behaviour from the Internet. ^30^ Oral sex among high school adolescents was 3840, according to a study in Addis Ababa, Ethiopia. Adolescents who had practiced oral sex is 5.4% (190). Oral sex is closely related to the concept of best friends (AOR = 5.7; 95% CI 3.6–11.2) and having illiterate mothers (AOR = 11.5; 95% CI 6.4–18.5). ^31^ Besides that, oral sex behaviour is one of the activities carried out and offered by sex workers. The percentage of women who engage in oral sex varies in some countries in Asia: 5% in Indonesia, ^32^ 16% in Thailand, ^33^ and 18% in India. ^34^ These numbers show that oral sex is not a new behaviour globally. Many of these behaviours are carried out in developing countries and sophisticated countries. The difference is that technological developments accompanied by freedom of expression make this behaviour frequently appear in pop culture.

## Perspectives and motives in oral sex

NBC (2005) reports that 40–47% of young people in the USA who do oral sex do so because they do not need to worry about pregnancy, meet the right person and feel the sense for the first time in adolescents and young adults. Other reasons, many couples in serious relationships want to experience sexual pleasure but are not pregnant, ^14^ experience sex without using a condom, ^14^ ^,^ ^35^ ^–^ ^37^maintain virginity, ^35^ ^,^ ^37^ answer curiosity about sexual activities, ^35^ and believe that oral sex has a low risk of STD transmission. ^14^ ^,^ ^35^ ^–^ ^37^

In teenagers, having oral sex is related to increased popularity, ^38^ improvements in relationships among teenagers, ^35^ ^,^ ^36^ engaging in sexual activity without commitment, ^14^ assumptions that oral sex is not sex, ^14^ and also sex with someone is a generous behaviour. ^38^ The same perspectives and motivations were also found in married couples. Holway and Tillman reported on the timing of oral sex in marriage among young adult couples in the USA. Their report stated that women who were late or never performed oral sex reported being more satisfied with their relationships with their partners. The fundamental reason is they do not feel forced when having sexual intercourse and do not need to worry about disease transmission due to sexual relations. ^39^ It is important to know that understanding who will perform and receive oral sex is a big deal among teenagers. The view of gender theory affects the perspective of teenagers in performing oral sex. Oral sex in both genders is said to be equal, but oral sex in women is a big deal, while this is not for men. ^40^ Maybe this is the reason that men are frequently given oral sex (fellatio) than perform oral sex (cunnilingus). ^24^

Meanwhile, oral sex is behaviour that risks transmitting various diseases. Teens and young adults claim oral sex is less risky and more acceptable than vaginal sex. Sometimes they perform oral sex because they do not want contraception and protection while having sex. ^41^ This fact shows the need for health workers and counsellors who provide the correct perspective in education or sex counselling. ^39^ ^,^ ^41^

Penhollow et al. also conducted a study on 408 students in the USA. They asked about students’ involvement in religious activities, frequency of worship attendance, their feelings about religion, and their perception of God in sex, and then looked at their participation in several sexual activities such as oral sex. ^42^ Results indicated that religiosity variables, mainly frequency of religious attendance and religious feelings, were significant predictors of sexual behaviour, such as oral sex. ^30^ The religious factor was reported as one of the reasons a person does not engage in oral sex. ^28^ Apart from that, another influencing factor is higher education. ^39^

## Attitudes toward oral sex

Attitudes towards oral sex tend to vary in the general population. The study surveyed 8600 people aged 16–64 years in Australia regarding their sexual activity. The analysis results stated that oral sex is one of the mandatory activities in sex activities besides kissing, cuddling and vaginal intercourse. About 74% of their respondents reported that women want stimulation over their genitals orally, and 70% stimulate their partners’ genitals orally. ^43^ This result proves that genital mouth stimulation is widespread in married couples. ^44^

Men at risk for adultery are more likely to engage in oral sex. ^45^ Oral sex can be used to detect infidelity, material retention behaviour and orgasm with sperm retention. Oral sex, used as a detector for adultery, is indicated, for example, in cunnilingus; one study states that men are still likely to feel and smell their rival’s semen around the vagina related to their previous sexual activity. ^46^

Many studies have reported that couples during oral sex mostly do not use condoms. However, Auslender et al. from USA reported different results. The results in young adults who took protective measures to reduce the process of transmitting diseases like *Herpes simplex* by using microbicide surrogate products. ^47^

A study at Northeastern University, USA, in 2015, enrolled 346 men and women regarding their expressions and attitudes towards oral sex, either receiving or doing it for the first time. Various expressions, including happy, fearful, indifferent, strange, disgusted, surprised, relieved, proud and sad, were addressed. The most common expression for oral sex recipients was feeling happy (38.7%). Interestingly, 10.8% thought this behaviour was disgusting, and 8.2% thought this was strange. In another oral sex study, the dominant feeling was fearfulness which was as much as 17.1%, 15.8% felt indifferent, 2.8% felt disgusted, and only 12% felt happy doing it. ^48^ Another report is a focus group discussion study involving women with fellatio and cunnilingus experiences. They stated a feeling of good emotional vulnerability when engaging in oral sex on their partners. ^49^

During oral sex, the aim is for the partner to orgasm. Richerts et al. (2006) conducted a study on a large population in Australia; 50% of women experience orgasm during vaginal intercourse, but when intercourse is added with cunnilingus activity, 73% will experience orgasm. Oral sex strengthens the increased incidence of orgasm in partners, especially in women. ^27^

## Oral sex risk

Many reports have stated that oral sex can cause local infections. The pathogens often transmitted through oral sex are viruses ( *Herpes simplex* viruses, Hepatitis virus, Human papillomaviruses, and less frequently HIV) and bacteria (mainly syphilis, gonorrhoea, group B Streptococcus). ^50^ ^,^ ^51^In addition, infections such as *Molluscum contagiousum*, Candidiasis *,* Epstein Barr virus and Aspergillosis can also be spread through the process of oral sex. ^52^

Gonorrhoea infection is possible in oral sex. Sex workers with inconsistent condom use for oral sex were 17.1 times more likely to develop pharyngeal gonorrhoea (95% CI: 8.0 ± 36.5) than consistent condom users. ^53^ HSV-1 is transmitted through genital and oral contact. This lesion is one of the most prevalent lesions among women, even in young women. ^54^ ^,^ ^55^ HSV-1 is more likely to spread to the female genital organs than male genital organs. ^56^ For young people, acquiring a lifelong recurrent infection such as genital herpes can invoke anxiety, guilt and social-sexual isolation and is an unfortunate surprise diagnosis. ^57^ ^,^ ^58^

Unusual infections have also been reported after oral sex. The incidence of pharyngitis with *Trichomoniasis vaginalis* was reported in men who routinely had oral sex with their partner who had vaginal Trichomoniasis. ^59^ It is infrequent for Trichomoniasis to spread by mouth and cause a local infection. Parasites such as *Toxoplasma gondii* is found in ejaculate fluid, possibly spread through oral sex. ^60^ ^,^ ^61^ There may be a positive association or association between Toxoplasma transmission and oral sex behaviour, especially in fellatio. However, this condition depends on fellatio activity and whether sperm is swallowed or not.

Froissart et al. has reported severe infection. They reported two cases of oral sex activity causing severe infection. The first case complained of fever, pubic erythema, penile oedema, and cellulitis and the second case complained of genital bubo, foreskin oedema, fever, glans erosion and cellulitis. Both of these cases occurred after performing fellatio activities with their sex partners. In this case, the suspected transmission process was abrasion of the penis during traumatic oral sex. Pubic cellulitis is an infrequent case. Genital organ trauma due to a partner’s bite is the presumed port of entry. ^50^

Lutz and Gerber (2020) also reported severe soft tissue infections such as Fournier’s gangrene through oral sex with commercial sex workers. This male patient experienced swelling and sloughing of the skin of his penis after reportedly engaging in oral sex. His partner nicked the shaft of the penis using her teeth during intercourse. ^62^ Takenouchi et al. also reported a rare infection, Lemierre syndrome, in a 58-year-old man after oral sex with a sexual partner two days previously. ^63^ Lemierre syndrome is characterised by thrombophlebitis of the internal jugular veins and bacteraemia caused by anaerobic organisms following a recent oropharyngeal infection. ^64^

Cunnilingus should be avoided in pregnant women. Hosseini and Hunt reported a case of *Streptococcus mitis* chorioamnionitis in a 43-year-old pregnant woman who had oral sex with her partner. This infection occurred 10 days before contractions after dental scaling and oral sex. ^65^ A similar case was also reported by Gherman et al. in Australia of an infection with *Streptococcus viridans* after cunnilingus. ^66^ Both *Streptococcus mitis* and *Streptococcus viridans* are normal flora in the oral cavity.

WHO guidelines for adolescent sexual and reproductive health and rights in 2018 recommend giving antibiotic prophylaxis to children who experience sexual abuse suspicion, one of which is oral sex. WHO considers that oral sex by an unknown offender has a significant risk of sexually transmitted disease. ^67^ Oral sex was a riskier behaviour compared to vaginal and anal sex according to Barbara et al. Cunnilingus has an OR of 2.199 and fellatio an OR of 2.756 for infection. This study reported that people who performed oral activities have higher risks associated with STD/HIV transmission than those who do not. ^68^

Other serious risks that arise due to oral sex behaviour include human papillomavirus infection, which causes oral squamous papilloma, oral verruca vulgaris, condyloma acuminata, focal epithelial hyperplasia, epidermoid carcinoma and oropharyngeal squamous cell carcinoma. ^69^ ^–^ ^71^ One study from Indonesia proves a strong correlation between oral and anal activity among homosexuals and HPV infection (OR 6.854). ^72^ Women who had oral sex more than three times with their partners in the previous month had a higher risk of developing oral HPV than those who had less. ^73^

## Oral sex benefits

Meuleman et al. reported in the Netherlands that oral sex might be a protective factor in recurrent miscarriage cases. This study was conducted on 97 women who experienced unexplained consecutive miscarriages. An imbalance of immunity in the embryo’s implantation into the endometrium causes recurrent miscarriage cases. ^74^ Koelman et al. from The Netherlands also stated that exposure to oral sex could reduce the incidence of preeclampsia. ^12^

Pittrof et al. studied 619 women who engaged in oral sex in the USA. They have a lower risk of developing endometriosis and pelvic inflammatory disease than their counterparts. Endometriosis and PID require adaptive immunity in the lymphoid system, leading to the assumption that oral sex can be a protective factor. ^75^ Meanwhile, the oropharynx is a channel that is rich in lymph channels, thereby facilitating the stimulation of adaptive immunity. Primed lymphocytes in the nasopharyngeal tract will be found later in the endocervix. ^76^ In addition, it is possible to induce genital T-cells and B-cells through sublingual immunisation. ^77^

Testosterone also affects the libido of women. Testosterone in semen can logically affect the libido of the sexual partner. Women who did not use condoms at the time of intercourse had more sexual intercourse than women who did. ^78^ There are no specific reports in the case of women who swallowed semen. Semen also had an antidepressant effect, such as serotonin. It has been reported that women whose partners used a condom during intercourse were more likely to be depressed than those who did not use it. ^78^

Opioids are some of the interesting substances that are also present in semen, such as endorphin, enkephalin and other cytokines. These substances affect sperm motility, such as endorphins and calcitonin. ^79^ Endorphin and enkephalins function to reduce anxiety and induce analgesia and drowsiness. ^80^ Likewise, oxytocin is a hormone that plays a role in increasing bonding and intimacy in partners, and this hormone impacts penile erection and female orgasm. ^81^ However, the activity levels of these substances will be significantly reduced in the blood due to the absorption, distribution and metabolism processes in semen ingestion.

Besides that, several benefits are felt during oral sex, such as satisfaction in intercourse and emotional or physical pleasure in intercourse. A study in the USA and Germany on 410 women who had oral sex showed that their male partners delay orgasm, so the intercourse time is longer. ^82^ Oral sex is a positive activity that provides sexual satisfaction and relationships. ^83^ ^,^ ^84^

Another benefit of oral sex is sperm retention by male partners. ^85^ Cunnilingus is considered one of the activities that help women experience orgasm. ^27^ Reports show that oral sex helps sexual activity last longer than usual. ^86^ Another study from the USA reported that 233 men who had oral sex with their partners would spend more time having sex with them, doing more copulatory behaviour that replaced semen, and reported greater sexual arousal. ^45^ Men who engage in oral sex with their partners as part of their strategy provide a more comprehensive range of benefits. Men who are higher in consideration are more likely to benefit their partner. ^86^

## The religious and moral issue on oral sex

The religious and moral issue on oral sex agrees that “bedroom activities” are private and personal matters. We must understand that sexual fantasies and bed activities are a social and moral responsibility. Sometimes it is hard to judge if a “certain bed activity” is right or wrong. If couples enjoy and accept each other, it is fine to choose their sexual preference. All need to return to the individual reason they chose and did that sexual activity.

The research we reviewed reported numerous oral sex activities in the USA and Australia. Still, very few in the Middle East and Asia as Western countries are known to be more liberal in their sexual behaviour. Some of the reasons and arguments are worth understanding, considering the perspectives, motivations and attitudes behind oral sex. It is used as a medium to enjoy and show affection for each other, but love and sex are two different things. Expression of love does not always end with sex. Sex is included in passion, which is only one part of the love component in the triangular theory of love. ^87^ The expression of love which is translated into sexual behaviour is not always justified, especially when it gives rise to preferences such as paraphilias. ^88^ Things like this are often found in married couples. However, not all couples accept all these behaviours. It is, therefore, necessary to re-communicate their respective sexual preferences.

Oral sex has long been known to clash with people’s moral code and religious values. Oral sex is not accepted in all circles, although it is an ancient sex behaviour. Religion is one of the factors that shape people’s cognition to consider whether something is right or not. ^30^ We will quote several religious views regarding oral sex. In Christianity, sex is sacred and full of commitment. ^89^ Sex life in the Bible is described a lot in the book “Song of Songs”; this section shows that sex is something intimate and sacred between couples. ^90^ Ed. Wheat and Gaye Wheat, in their book “Intended for Pleasure”, state that oral sex is not explicitly found in the Bible, but Wheat argues that back to the basic understanding that everything that is not good and inappropriate for the body is not worth doing. “Every human being is responsible for their own body to glorify God.” as written in 1 Corinthians 7:3–5 and Colossians 3:23. ^91^

In the Islamic religion, Chawki stated in his article that oral sex is a disgusting activity and should not be done, but some views state that this is not explicitly stated. ^92^ Another opinion said that oral sex is something that can be done as long as it is done in the corridor of husband and wife as written in Q.S. Al-Baqarah: 223. ^93^ Some views also state that married couple may pick any practical and mutually agreeable type of intercourse or other sexual activity except for those explicitly disallowed (such as anal intercourse, oral sex and sadomasochism). ^94^ In Buddhism, sexual misconduct is exemplified in the *Sutra of the Upsaka Precepts*, such as sex at the wrong time, wrong place, incorrect partner, virgin or another man’s wife; or if he engages in sexual self-gratification. ^95^ Values like this will affect cognition for each individual. We agree values like these are important in shaping their perception of (oral) sex. However, a strict understanding of religion also affects a person’s rigidity in understanding sex, so sometimes many people fall into disorders such as sexual aversion or sexual disgust because they view sex as a sin. ^96^

Everyone should have the right “sex education” to be more careful about having oral sex and understand that oral sex is not always safe. The oral sex that is done just for the sake of popularity and satisfying curiosity is not justified because the risks outweigh the benefits, especially for teenagers and young adults who are not married yet. It turns out that pop culture and technology are the factors that contribute more to oral sex behaviour, according to our analysis. We also find these factors in countries such as Nigeria and Ethiopia although the results of many studies are obtained in countries such as the USA and Australia. ^30^ ^,^ ^31^ So, parents are needed to direct and monitor teenagers about sex. ^97^

Sometimes, pleasure considerations should be put aside for couples rather than safety and health. If oral sex only aims to get pleasure, couples must understand that pleasure does not always have to be with sex, especially oral sex. Couples must understand that there is a “sensate focus”, an activity that can deliver pleasure without having sex. ^98^ On the other hand, the health benefits obtained through oral sex, such as immune modulation in preventing recurrent miscarriages and preeclampsia, deserve to be considered and even further investigated. In the end, oral sex was returned to the couple’s respective views with care.

## Conclusion

It is fascinating to discuss the various aspects of oral sex. The social aspect contributes to increasing the behaviour of oral sex activities. Technological developments accompanied by the penetration of pop culture have a significant impact on making people perform oral sex from year to year. This is very visible in the sexual activity of adolescent groups. The reasons they also perform oral sex are heavily influenced by pop culture factors that they find out in songs or films that feature the “hooking up culture.” However, a few refrain from having oral sex due to religious and moral views. In addition, the adolescent group still has the wrong understanding that oral sex is safer than vaginal intercourse, whereas oral sex increases the incidence of STIs among teens. Older people use oral sex as a medium to enjoy intimacy with their partner; oral sex is a variation of sexual behaviour to prevent boredom in a relationship. As a result, oral sex provides improved relationship quality if done with openness to each other, although oral sex has pros and cons based on religious views. On the other hand, the health benefits obtained through oral sex, such as immune modulation in preventing recurrent miscarriages and preeclampsia, deserve to be considered and even investigated further. In the end, oral sex returned to each couple’s respective views with care.

## Data availability

No data are associated with this article.
