# Supplementary material for: Framing and understanding the whole aspect of oral sex from social and health perspectives: a narrative review
Source: F1000Res. 2022 Jul 21;11:177. Originally published 2022 Feb 14. [Version 3] doi: 10.12688/f1000research.108675.3 (PMC9377381; doi:10.12688/f1000research.108675.3)
Supplement: Peer review history

# Peer review: Reviewer response for version 3

**DOI:** 10.5256/f1000research.136066.r144857

**Published:** 2022-07-22

**Authors:** Gina Anindyajati

**Affiliations:** 1 Department of Psychiatry, Faculty of Medicine, Universitas Indonesia-Cipto Mangunkusumo Hospital, Jakarta, Indonesia

I approve of the revised article.

Is the review written in accessible language?

Partly

Are all factual statements correct and adequately supported by citations?

Partly

Are the conclusions drawn appropriate in the context of the current research literature?

Partly

Is the topic of the review discussed comprehensively in the context of the current literature?

Partly

Reviewer Expertise:

NA

I confirm that I have read this submission and believe that I have an appropriate level of expertise to confirm that it is of an acceptable scientific standard.

# Peer review: Reviewer response for version 2

**DOI:** 10.5256/f1000research.121886.r140813

**Published:** 2022-06-27

**Authors:** Gina Anindyajati

**Affiliations:** 1 Department of Psychiatry, Faculty of Medicine, Universitas Indonesia-Cipto Mangunkusumo Hospital, Jakarta, Indonesia

Comprehensive discussion

- Introduction - the purpose of this review is not exactly aligned with the title of the manuscript. As a reader, we expected to develop understanding about oral sex activities from the social and health perspectives. However, in the introduction we were getting more information on the definition of oral sex. Probably, it will be more suitable if the authors emphasise more on the contrary view between many perspectives about oral sex, thus point out the importance of the authors review.
- Introduction -  considering the purpose of this study, will it be more appropriate if the authors make a comparison about view or trend of oral sex, before and after the technological advancement as well as the increase penetration of pop culture? This will need a specific time period as boundaries for the literature searching as well as the discussion part.
- Introduction - if the authors decided to write the comparison, it is also essential to write the effect of this difference in order to give a sense of urgency. For example, the authors can put how the increase of oral sex behaviours lead to higher incidence of STDs, oral cancer, etc. A paper from Kumar et al (2015) might be a start to look for further supporting evidence. ^1^
- Another specific part needed from this manuscript is regarding the scope. Since it is a global view, maybe we can make it more pointed for the title.
- Method - any inclusion or exclusion criteria for the articles used in this narrative? For example, language and years of publication?
- Results - write a statement about how the authors found their evidence; including how many papers the authors found, limitation and strength of their evidence.
- Discussion - please provide specific time references for pop culture. Will it be different now and then, or can we generalise all time periods of any pop culture product?
- Discussion - start with direct answer of the manuscript’s objective, followed by description of the authors finding and any relevant evidence.

Factual statements & citation

- Introduction - Is there any supporting evidence for this claim? But currently, the trend of oral sex has increased prominently. The development of technology and media also supports this.
- Reference from Lewis & Marston can be used to support the evidence from young adults perspectives, in regards to social narrative. ^2^
- Results & Discussion - Is there any supporting evidence for this claim? the difference is that technological developments accompanied by freedom of expression make this behaviour a lot of popping up in the population
- Discussion - since the authors would like to explain the role of pop culture, it will be helpful for the reader to get a description on how pop culture is spread worldwide. Whether there was any difference due to licence, law, or any public policy, and maybe the country’s perspective about sexual content on any entertainment forms.

Language

- The manuscript is provided with clear definition about oral sex, however it will be helpful if the authors write about what they meant about pop culture, social as well as health views in the introduction part.
- The manuscript is written in English by using the grammar of Indonesian language. It will be easier to understand the objective and main point of this article, if this manuscript is being consulted with a formal service for academic writing.

Conclusions

- Future direction & Conclusion - this part was enriched by the religious view, unfortunately the view was not discussed on the result & discussion section. The authors need to rearrange the structure of their paragraph, so it will be coherent and in line with the objective.

Future direction & conclusion shall be started with direct answer of the manuscript’s objective.

Is the review written in accessible language?

Partly

Are all factual statements correct and adequately supported by citations?

Partly

Are the conclusions drawn appropriate in the context of the current research literature?

Partly

Is the topic of the review discussed comprehensively in the context of the current literature?

Partly

Reviewer Expertise:

Psychosexual; including sexual identity and behavior in psychiatric field

I confirm that I have read this submission and believe that I have an appropriate level of expertise to confirm that it is of an acceptable scientific standard, however I have significant reservations, as outlined above.

## References

[1] : Oral sex and oral health: An enigma in itself. *Indian J Sex Transm Dis AIDS* .36(2) : 10.4103/0253-7184.167133 129-32 10.4103/0253-7184.167133 26692602PMC4660550

[2] : Oral Sex, Young People, and Gendered Narratives of Reciprocity. *J Sex Res* .53(7) : 10.1080/00224499.2015.1117564 776-87 10.1080/00224499.2015.1117564 26849152PMC4960515

# Response

**Published:** 2022-07-11

**Authors:** Cennikon Pakpahan

**Affiliations:** Airlangga University, Indonesia

Thank you very much for your valuable opinion on our paper. 

 1. We added some further explanations in the introduction, reinforcing the ideas conveyed through this article. What are the social and health aspects that are the pros and cons of oral sex? We reaffirm the purpose of this review according to the title. In addition to the introduction, we added supporting articles according to the reviewer’s suggestions.

 2. We removed the method, result, and discussion sections to avoid confusion/misunderstanding of this type of article. Following the title, the approach we use in writing this article is a narrative review (literature review), which is undoubtedly different from a systematic review. Narrative review does not require a particular protocol or criteria for articles to be included in the review, as Ferrari (2015) stated in his article, “Writing narrative style in literature reviews”. So, in particular, we do not have a systematic report on how many articles were by the theme we conveyed, like a systematic review.

 3. We added narration on oral sex and pop culture, according to the reviewer’s suggestion. The explanation of the influence of pop culture deserves attention. The extent to which pop culture has an impact on oral sex is also added.

 4. We have added some suggestions regarding citations to provide strong evidence for our paper.

 5. We proofread the language according to academic needs.

 6. In the future directions and conclusion session, we changed it to “religion and moral issue in oral sex.” While the conclusion section we made at the end and written according to the article’s purpose at the beginning, as suggested by reviewers.

 We wait for good news from you. 

 Best Regards

# Peer review: Reviewer response for version 2

**DOI:** 10.5256/f1000research.121886.r138176

**Published:** 2022-05-30

**Authors:** Gajanan S. Bhat

**Affiliations:** 1 Department of Urology, Andrology and Sexual Medicine, TSS Shripad Hegde Kadave Institute of Medical Sciences, Sirsi, Karnataka, India

Though I appreciate the authors for their excellent effort to address an important issue such as oral sex, the stated aim in the introduction in no way matches the description and hence there is a total disconnect with the stated aim and the details in the article.

 Another important shortcoming of the article is the methodology. Whether you call the review a narrative or otherwise, the details of methodology and the literature extracted for review are too little to call it a review. Because of these shortcomings, I cant accept this as a review. It can at best be called a short communication or a letter to the editor. Kindly go through the following referenced article for improving the manuscript ^1^.

Is the review written in accessible language?

Partly

Are all factual statements correct and adequately supported by citations?

Partly

Are the conclusions drawn appropriate in the context of the current research literature?

No

Is the topic of the review discussed comprehensively in the context of the current literature?

No

Reviewer Expertise:

Sexual Medicine

I confirm that I have read this submission and believe that I have an appropriate level of expertise to state that I do not consider it to be of an acceptable scientific standard, for reasons outlined above.

## References

[1] : Checklists for review articles. *BMJ* .1994;309(6955) : 10.1136/bmj.309.6955.648 648-51 10.1136/bmj.309.6955.648 8086990PMC2541456

# Response

**Published:** 2022-05-30

**Authors:** Cennikon Pakpahan

**Affiliations:** Airlangga University, Indonesia

Dear Reviewer,  

 Thank you for your valuable review for our paper, but there are some things we need to clarify regarding the methods and literature suggestions the reviewer provides.

 We take a narrative review approach instead of a systematic review by the type of approach we take on this issue. Refer to some literature related to the definition of this Narrative review. *Narrative Review or Narrative Overview or Literature Review is the type first-year college students often learn as a general approach. Its purpose is to identify a few studies that describe a problem of interest. Narrative reviews have no predetermined research question or specified search strategy, only a topic of interest. They are not systematic and follow no specified protocol. No standards or protocols guide the review. Meanwhile, the literature suggestion you gave is an approach in a systematic review, not a narrative review.* We think that the two are different.

 Therefore, because we wrote this in the form of a narrative review, we have followed the writing protocol as recommended https://www.sciencedirect.com/topics/psychology/narrative-review

 Here are some references that we include to explain the types of articles we write.

 1. Ferrari, Rossella. Writing narrative style literature reviews. 2015. Medical Writing Vol. 24. DOI: 10.1179/2047480615Z.000000000329

 2. Green, Bart, Johnson, Claire, Adams, Alan. Writing narrative literature reviews for peer-reviewed journals: secrets of the trade. 2001. JOURNAL OF CHIROPRACTIC MEDICINE. available from: https://familymedicine.med.wayne.edu/mph/project/green_2006_narrative_literature_reviews.pdf

 For your suggestion about the aims and the explanation, we will try to improve it. 

 Best Regards,

# Response

**Published:** 2022-07-11

**Authors:** Cennikon Pakpahan

**Affiliations:** Airlangga University, Indonesia

Thank you for your valuable opinion on our paper.

 We try to revise the paper according to your suggestion. 

 We wait for the good news.

# Peer review: Reviewer response for version 2

**DOI:** 10.5256/f1000research.121886.r126698

**Published:** 2022-03-10

**Authors:** Thi Tu An Nguyen

**Affiliations:** 1 School of Social and Political Science, Monash University, Clayton, VIC, Australia

Dear authors, 

 Thank you for quickly revising this article as I suggested. It is much better now. 

 Good luck,

 An

Is the review written in accessible language?

Yes

Are all factual statements correct and adequately supported by citations?

Partly

Are the conclusions drawn appropriate in the context of the current research literature?

Partly

Is the topic of the review discussed comprehensively in the context of the current literature?

Partly

Reviewer Expertise:

sexual and reproductive health, disability, applied health, sociology, anthropology, family and gender.

I confirm that I have read this submission and believe that I have an appropriate level of expertise to confirm that it is of an acceptable scientific standard.

# Response

**Published:** 2022-03-10

**Authors:** Cennikon Pakpahan

**Affiliations:** Airlangga University, Indonesia

Thank you for your very meaningful input to improve our article.

# Peer review: Reviewer response for version 1

**DOI:** 10.5256/f1000research.120083.r123285

**Published:** 2022-02-15

**Authors:** Thi Tu An Nguyen

**Affiliations:** 1 School of Social and Political Science, Monash University, Clayton, VIC, Australia

First, I would like to take this opportunity to say thank you for giving me this chance to review this interesting topic.

 In my opinion, I would like to say thank you to the authors who spent time writing this article. However, this is not a review paper, because:

- There is no rationale for this paper. Why did you do this review? What contexts are you looking for? Are you looking for this in a certain country or globally?
- Method: what databases did you search for these papers? Time frame? Countries? Etc?
- The topic of the review is not discussed comprehensively in the context. To me, this paper is very descriptive. It needs more critical thinking.
- The conclusion is so simple. The authors should restate their aims/objectives and future directions or implications (if needed).

I think this paper should be revised a lot before it can be published. My recommendations are below:

**Introduction:**

 The authors need to clarify: Why are you doing this review? What are your aims/objectives?

**Defining oral sex and pop culture**

 Paragraph No3 (from the top) - page 3/9: The last sentence needs references.

 Paragraph No4 (from the top) - page 3/9: The first sentence needs references.

**Prevalence of oral sex**

 I suggest this headline should be ***The prevalence of oral sex of some countries around the world.***

 This section is so descriptive. You should say some things such as what is your thinking of these oral sex rates?

**Perspectives and motives in oral sex**

 You should add on the countries where these studies were conducted. Again, what do you think about these studies influencing your aims?

**Attitudes in oral sex**

 Again, which nationalities of these participants in these studies?

 Paragraphs No 2&3 should be deleted because they are irrelevant to this section.

**Oral sex risk**

 This section is good.

**Benefits obtained due to oral sex**

 I suggest this headline should be “oral sex benefits”.

 Again, which nationalities of these participants in these studies?

Is the review written in accessible language?

Yes

Are all factual statements correct and adequately supported by citations?

Partly

Are the conclusions drawn appropriate in the context of the current research literature?

Partly

Is the topic of the review discussed comprehensively in the context of the current literature?

Partly

Reviewer Expertise:

sexual and reproductive health, disability, applied health, sociology, anthropology, family and gender.

I confirm that I have read this submission and believe that I have an appropriate level of expertise to confirm that it is of an acceptable scientific standard, however I have significant reservations, as outlined above.

# Response

**Published:** 2022-02-26

**Authors:** Cennikon Pakpahan

**Affiliations:** Airlangga University, Indonesia

We are very grateful for the excellent feedback from reviewers. We have considered it and revised it according to your suggestions in our latest version. We are waiting for a good response from you.
